# Effect of Statin Therapy on the Plasma Concentrations of Retinol, Alpha-Tocopherol and Coenzyme Q10 in Children with Familial Hypercholesterolemia

**DOI:** 10.1007/s10557-020-07091-w

**Published:** 2020-10-14

**Authors:** Radosław Motkowski, Mateusz Maciejczyk, Marta Hryniewicka, Joanna Karpińska, Bożena Mikołuć

**Affiliations:** 1 Department of Pediatrics, Rheumatology, Immunology and Metabolic Bone Diseases, Medical University of Bialystok, ul. Waszyngtona 17, 15-274 Bialystok, Poland; 2 Department of Hygiene, Epidemiology and Ergonomics, Medical University of Bialystok, 15-274 Bialystok, Poland; 3 Faculty of Chemistry, University of Bialystok, 15-274 Bialystok, Poland

**Keywords:** Familial hypercholesterolemia, Simvastatin, Children, Retinol, Alpha-tocopherol, Coenzyme Q10

## Abstract

**Purpose:**

Familial hypercholesterolemia (FH) requires early treatment. However, statins, which are regarded the first-line therapy, have an influence on redox balance. Antioxidant vitamins are important for many metabolic processes in the developing body. There are few data available on the long-term safety of statin use in children. The aim of this study was to evaluate the influence of statin treatment in children with FH on plasma concentrations of antioxidant vitamins: retinol, alpha-tocopherol and coenzyme Q10.

**Methods:**

The first study group consisted of 13 children aged 10–18 years treated with simvastatin for at least 6 months, and the second group comprised 13 age- and sex-matched children with hypercholesterolemia, in whom pharmacological treatment had not been applied yet. Analyses were performed using a high-performance liquid chromatograph coupled with a MS detector.

**Results:**

The analysis did not reveal significant differences in the concentration of retinol, alpha-tocopherol or coenzyme Q10 between the studied groups. The adjustment of the concentrations of the vitamins to the cholesterol level also indicated no significant differences. We found no deficits in antioxidant vitamins in patients treated with statins, or any risk of adverse effects associated with an increase in their concentration.

**Conclusion:**

There is no rationale for additional supplementation using antioxidant vitamins or modification of low-fat and low-cholesterol diet in pediatric patients treated with statins.

**Electronic supplementary material:**

The online version of this article (10.1007/s10557-020-07091-w) contains supplementary material, which is available to authorized users.

## Introduction

Familial hypercholesterolemia (FH) is an autosomal-dominant genetic disorder characterized by an elevated low-density lipoprotein (LDL) cholesterol level and premature coronary heart disease due to atherosclerosis. The genetic cause for FH is most often mutation within the LDL receptor and apolipoprotein B genes. According to the study by Nordestgaard and Benn, the prevalence of heterozygous FH in Europe is 1 in 217 [1]. In FH patients, cardiovascular mortality is approximately 100 times greater than in the general population [2, 3]. Once the patient has been diagnosed with FH, they should follow healthy lifestyle advice and undergo lipid-lowering treatment [4, 5].

The most frequently used lipid-lowering drugs are statins. They are commonly used among adults, and they are efficient not only in lowering LDL and total cholesterol levels, but also in reducing the progression of atherosclerosis and the risk of premature coronary events [6–8]. The safety of these medicines in adults has been determined in a number of randomized trials of primary and secondary prevention of cardiovascular diseases [9], although there are also reports that dispute their effectiveness [10].

Fewer data are available on treating FH patients at developmental age with statin preparations. A meta-analysis of nine randomized and placebo-controlled studies involving a total of 1177 children was carried out in a study for the Cochrane Library [11]. High efficacy of statin preparations was demonstrated in children with FH, enabling on average a 30% reduction in LDL concentration (range 23–40%). No influence of statin treatment on body height or maturation was observed in this group, and no changes in liver enzymes, creatinine kinase or renal function were detected. In published observational studies on 186 Dutch [12] and 135 British [13] children treated with statins, a positive influence on lipid profiles was also found, and no significant adverse effects were revealed.

The safety of statin use in children has been assessed most often only in the context in which adverse effects in adults were previously described. It is known that the use of pharmacotherapy to reduce risk factors rather than for treatment of existing disease must be undertaken only when there is a low risk of harm. Therefore, despite important data on the ability of statins to lower the concentration of total cholesterol and LDL, the effectiveness of this type of treatment in the prevention of atherosclerosis and the safety of its long-term use with respect to the development and maturation of various systems and organs remain open questions in the pediatric population [11].

In the multifactorial pathogenesis of atherosclerosis, lipid accumulation, chronic inflammatory processes and oxidoreductive balance disturbances are of fundamental importance. Under normal conditions, there is harmony between oxidants and antioxidants. An increase in the production of free radicals or a decrease in antioxidant activity causes an imbalance towards the oxidation reaction, which is called oxidative stress. Protection against oxidative stress includes scavengers of reactive oxygen species. In the aqueous environment, these are vitamin C, uric acid and glutathione, and in the lipophilic environment, vitamin E, retinol, carotenoids and coenzyme Q10 (CoQ10) [14]. Vitamin A affects the inflammation and oxidant/antioxidant balance, and vitamin E is involved in lipid disorders and antioxidant processes. CoQ10 is to a large extent an endogenous metabolite which serves, inter alia, to restore vitamin A and E resources, and its synthesis is directly blocked by statin preparations.

Vitamin A in the human body comes from animal (retinol) or plant (carotenoids as provitamins) food sources. The liver contains most of the stored retinol, and this organ is responsible for its proper concentration in the blood. The biological activity of vitamin A is associated with the direct effect of retinol, its metabolite and transport proteins in the body [15]. Retinol circulates in serum in combination with retinol-binding proteins (RBP4) and later also transthyretin (TTR), and is freely converted into retinal and retinoic acid depending on the metabolic demand [16, 17]. Studies have demonstrated an association between the RBP4 concentration and inflammatory markers, insulin resistance, preclinical atherosclerosis and cardiovascular disease risk [18]. It was also reported that the expression of the CD36 scavenger receptor on macrophages is increased by free RBP4 and retinol-bound RBP4 [19]. This receptor is important in the initial stage of “foam cell” formation through internalization of oxidatively modified LDL, which initiates atherosclerotic plaque creation.

Vitamin E is a mixture of eight chemicals, four tocopherols and four tocotrienols. In plasma, it is transported mainly by lipoproteins. A total of 80–90% of all tocopherols in the blood are alpha-tocopherol. Vitamin E belongs to the nonenzymatic antioxidant defense system, whose main function is to inhibit lipid peroxidation. Peroxide radicals react 120 times faster with vitamin E than with polyunsaturated fatty acids [20]. As a result of this reaction, inactive tocopherol radicals are formed. In this way, vitamin E plays an important role in stabilizing cell membranes. It exerts an influence by transcription factor gene transcription or enzyme and molecular activity involved in cell signaling pathway regulation [14].

CoQ10 is a very powerful antioxidant in its reduced form. It is the only known fat-soluble antioxidant whose oxidized form can be reduced by the body's enzymatic systems and not by another antioxidant compound. Apart from its role in the energetic processes of cells, CoQ10 in the mitochondrial respiratory chain regenerates oxidized forms of other antioxidants [20]. CoQ10 biosynthesis is controlled by 3-hydroxy-3-methylglutaryl-coenzyme A reductase (HMG-CoA reductase), an enzyme that participates in the endogenous synthesis of cholesterol, which is directly inhibited by statin preparations. The inability to balance the reduced endogenous synthesis of CoQ10 through dietary supply leads to its deficit [21]. A congenital defect in the biosynthesis of CoQ10 is associated with a progressive decrease in muscle strength and disorders of central nervous system function in early childhood [22].

Observational studies have shown an inverse correlation between the intake of vitamins C and E and retinol and the occurrence of cardiovascular diseases [23]. The rates of mortality due to cardiovascular disease and cancer were also found to be lower in adults with higher serum concentrations of these vitamins [24]. Unfortunately, attempts at vitamin E supplementation in large randomized studies did not achieve the expected reduction in mortality rates [25]. The results of studies conducted in 29,000 adults in Finland, which showed that vitamin E and beta-carotene supplementation as a precursor of vitamin A was associated with an 8% increase in mortality and 18% higher incidence of lung cancer, aroused great concern [26]. Unfavorable results of interventions with beta-carotene and retinol were confirmed in subsequent studies [27]. More optimistic are the reports on the combination of procedures, i.e. statin therapy and antioxidant vitamin supplementation. The Heart Protection Study showed that simvastatin significantly reduced morbidity and mortality associated with cardiovascular diseases, and no additional risk was found with the use of antioxidant vitamins. Unfortunately, no further benefits were demonstrated [28].

A congenital metabolic disorder such as FH requires early intervention to reduce the risk of complications [29]. The therapy of first choice is treatment with statins, but there are few data on the long-term safety of their use in children. Oxidative stress plays an important role in the pathogenesis of cardiovascular diseases caused by atherosclerosis. The aim of this study was to evaluate the effects of statin treatment in children with FH on plasma concentrations of antioxidant vitamins: retinol, alpha-tocopherol and CoQ10.

## Materials

The study included children with FH diagnosed on the basis of molecular tests (described in Methods) and/or clinical criteria (high LDL level, family history of hypercholesterolemia or premature cardiovascular disease events) [30] after excluding secondary causes of elevated blood cholesterol levels. The exclusion criterion in both groups was the presence of systemic disease, including autoimmune, infectious, gastrointestinal, metabolic, pulmonary or cancer diseases, as well as clinical and laboratory signs of acute infection. The first group consisted of 13 children aged 10 to 18 years treated with simvastatin at a dose of 10 mg once daily for at least 6 months, and the second group comprised 13 children, matched by gender and age, in whom pharmacological treatment had not yet been applied. The diagnosis was confirmed molecularly in 10 children (77%) in the group treated with statins and in eight children in the group with hypercholesterolemia without statin treatment (62%). All children under study were on a low-cholesterol diet for at least 6 months before and during the study. Children taking vitamins and food supplements for the prior 3 months were excluded from the study. Children were under the constant care of the Metabolic Disorder Clinic of the University Children’s Hospital in Bialystok. In the group of children with hypercholesterolemia, a subgroup of four girls aged 12–18 years was determined for whom it was possible to assess changes in the plasma concentration of antioxidant vitamins before and after statin therapy.

The study was approved by the Bioethics Committee of the Medical University of Bialystok. The parents of the children included in the study gave their informed consent. The study was performed thanks to a grant from the Medical University of Bialystok (no. N/ST/ZB/16/001/1126).

## Methods

### Sample Preparation

Blood samples for testing were taken in the morning, at least 12 h after the last meal. The 1.8-ml samples for measuring the concentration of antioxidant vitamins were collected in test tubes with 3.8% sodium citrate in a 9:1 ratio. After spinning for about 30 min at 4 °C, the supernatant underwent deep freezing at −70 °C [31].

### Molecular Tests

Molecular tests were performed using the polymerase chain reaction restriction fragment length polymorphism (PCR RFLP) method and multiplex ligation-dependent probe amplification (MLPA) sequencing, and in some patients, with next-generation sequencing (NGS) on the MiSeq platform (Illumina) using the ADH MASTR (Multiplicom) kit. Tests were performed in the University Clinical Center of the Medical University of Gdańsk as part of the routine diagnostic procedure in patients with hypercholesterolemia.

### Determination of Plasma Lipophilic Antioxidants

Determination of plasma vitamin A (all-trans-retinol), vitamin E (L-tocopherol) and CoQ10 was performed using a high-performance liquid chromatograph (HPLC) coupled with a mass spectrometry (MS) detector equipped with a triple quadrupole (Shimadzu LCMS-8040). The ionization was conducted using the APCI [atmospheric pressure chemical ionization] mode, and data were processed using Shimadzu LabSolutions LCMS software.

Compounds were separated using an analytical column from Phenomenex (Kinetex XB-C18 100A; 50 mm × 3.0 mm, 1.7 um). The mobile phase consisted of isocratic solvent A (methanol; 0.01–2 min) and then isocratic solvent B (methanol/n-hexane, 72:28, *v*/v; 2.5–6 min). The temperature of the column was 40 °C. The flow rate was 0.4 mL/min, and sample injection volume was 10 μL. The settings and method for acquisition were optimized by the infusion of a 10-μg/mL solution of each compound. The APCI temperature was 350 °C, and the ion current was 4.5 μA. The flow of the drying gas (N_2_) was 10 L/min and that of the nebulizing gas was −3 L/min. The desolvation line (DL) and heat block temperature was 230 °C. All analytes were detected by MS/MS-multiple reaction monitoring (MRM) with unit resolution at both Q1 and Q3 (Table 1).

**Table 1:** The MS conditions for generation of positive ions of the analytes

Compound	Precursor ion (m/z)	Product ions (m/z)	Collision energy [eV]
Retinol	269.10	213.20 93.10	−12 −23
α-Tocopherol	429.30	165.10 137.05	−25 −48
Coenzyme Q10	863.60	197.15 109.10	−45 −47

### Statistical Analysis

The obtained results underwent statistical analysis in which, for quantitative variables, the arithmetic mean, median and standard deviation (SD) were calculated. The examined variable distribution was assessed by the Kolmogorov–Smirnov test. Because the tested variables were inconsistent with normal distribution, the Mann–Whitney *U* test was used. Spearman’s method was applied for assessing correlations between the variables. In calculations, a relevance level of *p* < 0.05 was accepted as statistically significant, authorizing the rejection of individual null hypotheses. The data were processed using the Polish version of Statistica 12.0 statistical software for Windows.

## Results

**Fig. 1:**
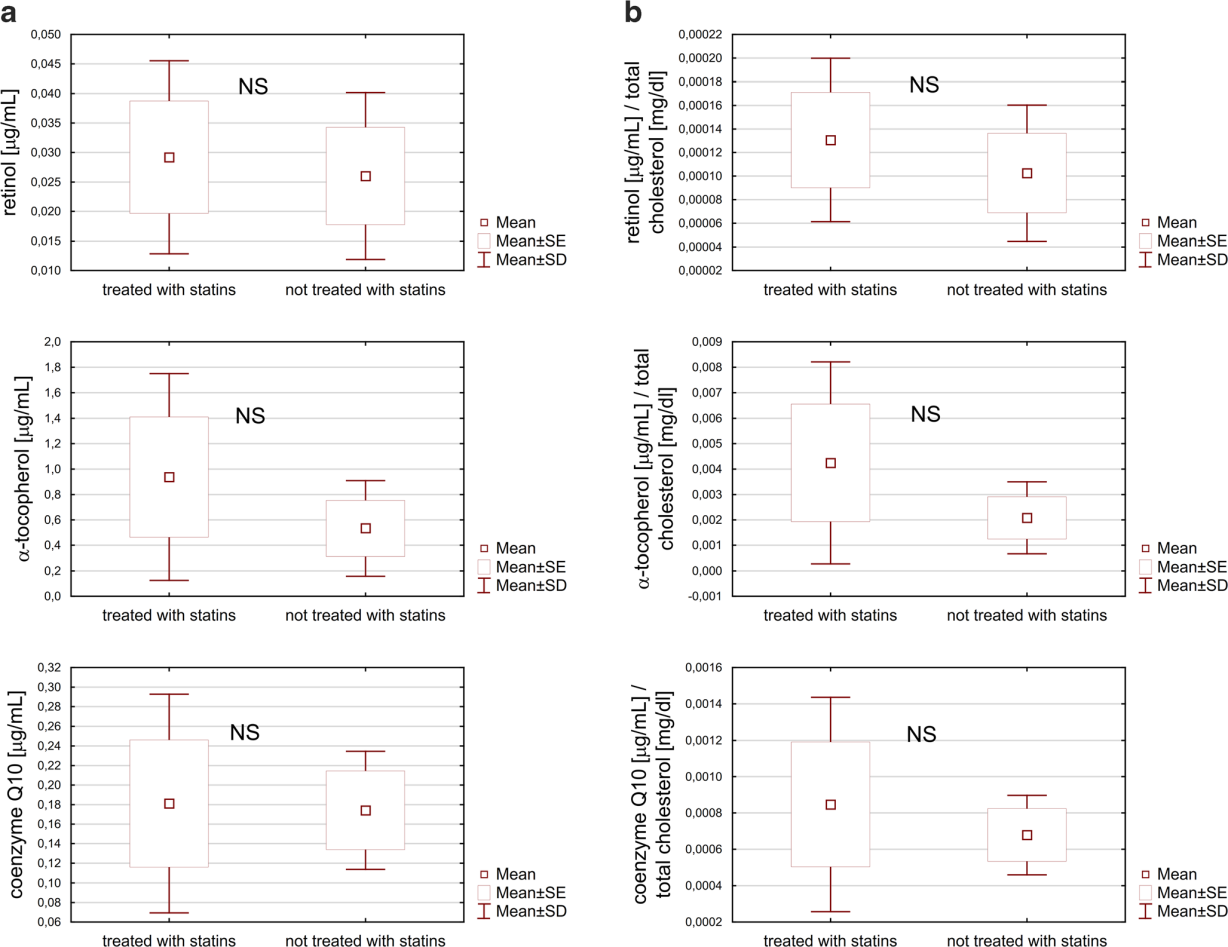
The concentration of antioxidant vitamins in plasma of children undergoing treatment with statins (*n* = 13) and not treated with statins (*n* = 13) assessed directly (**a**) and in relation to total cholesterol (**b**)

The detailed characteristics of the studied groups are presented in Table 2. The analysis did not reveal statistically significant differences in the concentration of retinol, alpha-tocopherol or CoQ10 between the groups of children with hypercholesterolemia (Table 3, Supp. data). A graphical presentation of the results confirms that there are no differences in the concentrations of retinol or CoQ10 between the groups of children with hypercholesterolemia (Fig. 1), whereas a tendency towards higher alpha-tocopherol concentrations was observed in the group of pharmacologically treated children compared with the group of children not treated with statins (Fig. 1). The relation between the concentration of tested vitamins and the concentration of total cholesterol also did not indicate any statistically significant differences in the range of parameters examined (Fig. 1, Table 4 – Supp. data).

**Table 2:** Characteristics of hypercholesterolemic groups (data presented as means and standard deviations where applicable)

	Treated with statins (*n* = 13)	Not treated with statins (*n* = 13)	*p*
Age [years]	14.03 (1.89)	13.59 (1.86)	0.511
Sex	9 girls, 4 boys	9 girls, 4 boys	
Family history of hypercholesterolemia			
Siblings	10 (77%)	5 (38%)	
Parents	13 (100%)	13 (100%)	
Grandparents	10 (77%)	8 (62%)	
Mutation status			
- LDL-R	7 (54%)	5 (38%)	
- APO-B	3 (23%)	3 (23%)	
- Not LDL or APO-B	1 (8%)	1 (8%)	
- Not performed	2 (15%)	4 (31%)	
Weight [kg]	54.1 (12.58)	57.1 (13.62)	0.488
Height [m]	1.6 (0.09)	1.6 (0.08)	0.840
BMI [kg/m^2^]	20.6 (3.54)	21.7 (4.21)	0.511
Systolic blood pressure [mmHg]	116.2 (9.47)	105.9 (30.44)	0.347
Diastolic blood pressure [mmHg]	72.1 (9.79)	71.8 (9.37)	0.852
Heart rate [bpm]	80.4 (16.65)	84.2 (14.5)	0.458
Glucose [mg/dl]	87.3 (8.07)	94.8 (9.05)	*0.014
Total cholesterol [mg/dl]	220.8 (28.81)	257.4 (26.84)	*0.003
HDL cholesterol [mg/dl]	63.5 (18.58)	56.4 (12.96)	0.418
LDL cholesterol [mg/dl]	143.9 (25.68)	186.1 (26.89)	*0.001
Triglyceride [mg/dl]	65.6 (18.35)	74.8 (23.86)	0.362

* statistically significant

The correlation evaluation revealed significant directly proportional dependencies between the concentrations of tested vitamins, i.e. retinol and alpha-tocopherol (*r* = 0.617, *p* < 0.001), retinol and CoQ10 (*r* = 0.483, *p* < 0.005) and tocopherol and CoQ10 (*r* = 0.797, *p* < 0.001).

**Fig. 2:**
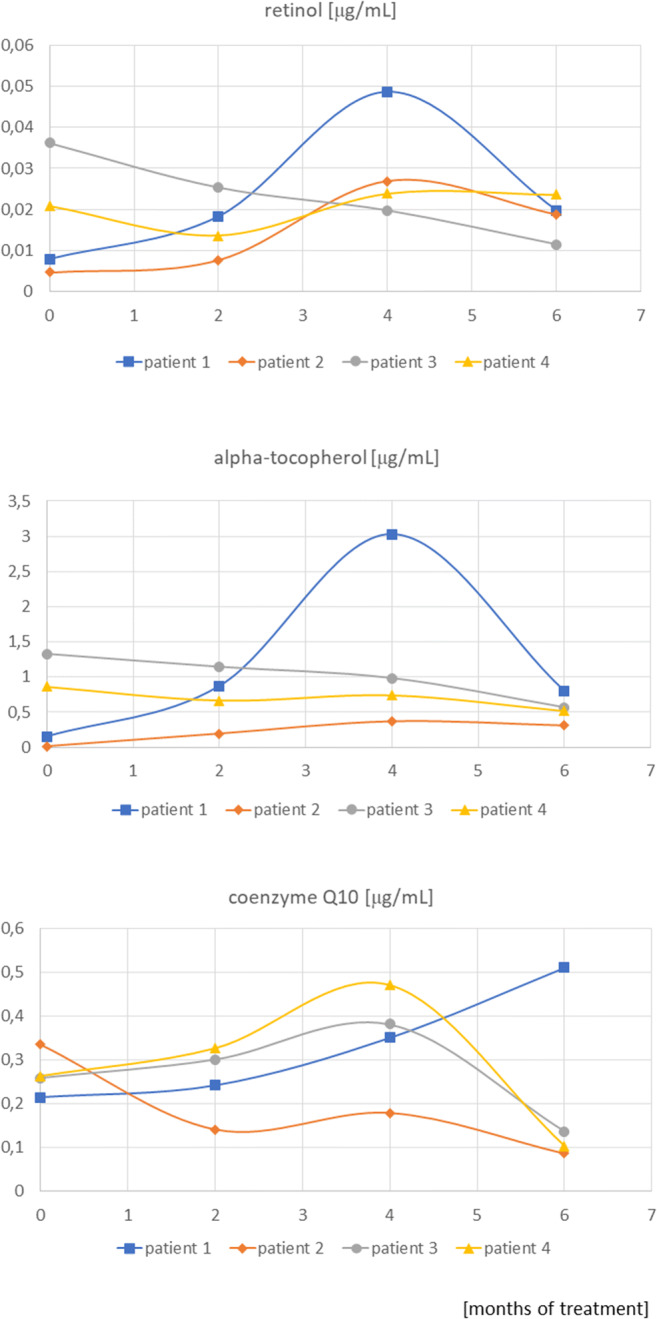
Changes in retinol, alpha-tocopherol and coenzyme Q10 concentrations were assessed during 6 months of treatment with statins in a subgroup of four patients

The analysis of changes in the concentration of antioxidant vitamins in the subgroup of four girls treated with statins did not indicate clear common trends (Fig. 2). The reduction in total cholesterol concentration in this subgroup was 23, 20, 24 and 20%, respectively, and that for LDL cholesterol was 33, 31, 39 and 37%. The relation of antioxidant vitamin concentrations to total cholesterol and LDL concentrations did not change the previously observed individual dependencies.

## Discussion

Familial hypercholesterolemia is a rare disease, and is diagnosed less frequently in asymptomatic children than in adults. The potential for prophylactic use of statins is very good, but any intervention in the developing body requires careful monitoring. Changes in the concentration of antioxidant vitamins have been shown in many physiological states and chronic diseases in humans. They have been repeatedly demonstrated in adults with common acquired diseases, such as diabetes or hypertension [14].

Vitamin A (retinol), by significantly modifying the expression of many genes, affects the growth in the prenatal and postnatal period, the efficiency of epithelia and the activity of the immune system [17]. In studies by Tonstad and Aksnes [32], in 151 children with FH not treated with statins, the concentrations of retinol and alpha-tocopherol were found to be comparable to those in healthy children. Statistical analysis showed a dependence of retinol concentration on the degree of maturation, sex and triglyceride concentration. In the same study, vitamin E concentration correlated only with total cholesterol concentration, which is consistent with observations from other studies [33].

There are few reports on the effect of statins on retinol concentration. Most available studies include beta-carotene as a vitamin A provitamin. In a 2007 report [34], treatment with simvastatin for 8 weeks in 76 adult patients with hypercholesterolemia was associated with an increase in retinol levels, but only cholesterol-adjusted data were reported. There are no studies in the available literature regarding the effects of statin therapy on retinol levels in children with hypercholesterolemia. In our study, treatment for 6 months did not significantly affect the concentration of this vitamin in the blood. No differences were found in the retinol concentration with respect to changes in total cholesterol concentration, despite a significant reduction in the concentration of the latter in the blood.

The direct influence of statin therapy on vitamin E concentration has been assessed in adults in several studies, and the results of these studies were summarized in a meta-analysis in 2015 [35]. In 13 selected studies, vitamin E concentrations were assessed in patients with various diseases and states (hypercholesterolemia, diabetes, renal disease and high risk of coronary artery disease) without treatment, after statin therapy and after statin therapy with vitamin E supplementation in different doses. Half of the studies included only men. The doses and preparations of statins also differed. The studies included a total of 1319 participants, of which 584 were treated with statins and 630 were in control groups. Most of the studies lasted up to 3 months, with the three longest studies conducted for 6, 12 and 36 months. A meta-analysis of these reports confirmed a significant decrease in the vitamin E serum concentration in adult patients treated with statins. At the same time, it was shown that the concentration of vitamin E in relation to the level of total cholesterol was higher in the group of patients treated with statins than in the control groups [36, 37].

The only available interventional study included in the meta-analysis, which comprised teenagers treated with lovastatin at a dose of 10 to 40 mg/day, also demonstrated a decrease in the vitamin E concentration in serum [38]. This study was the longest among those included, lasting 12 months. There were no data on vitamin E concentration with respect to total cholesterol in that study, but it must be stressed that the difference in direct vitamin E concentration before and after treatment was lower than all other studies with a shorter time span and among adults. In our study, no differences were found in either in the directly assessed plasma concentration of vitamin E or the concentration of vitamin E in relation to total cholesterol between the groups treated with statins and those not treated. However, the tendency towards higher alpha-tocopherol concentrations among patients treated with statins (Fig. 1) is noteworthy.

Vasankari et al. [39] observed that the decrease in the concentration of antioxidant vitamins described after treatment with statins in short-term studies disappeared with longer duration of treatment. In the abovementioned Korean studies with simvastatin, a dose-dependent effect of the drug on the antioxidant barrier measured by the trapping antioxidant potential (TRAP) parameter was demonstrated—slight at 20 mg, and significant at 40 mg [34]. During our study, statins were applied for a relatively long period and in only one dose, which was not modified during observation and was the same for all the patients.

There are also reports of possible interactions of statins with vitamin E at the level of expression of different genes. Vitamin E can enable genes by activating the pregnane X receptor (PXR) [40]. Statins also affect the activation of the PXR [41], which may explain the dose-dependent differences in the reduction of serum levels of vitamin E.

In a very comprehensive review of the literature conducted in 2015 [42], data on vitamin E concentration were evaluated from 132 studies involving 250,000 subjects. It was globally assessed that 13% of the subjects had very low concentrations of vitamin E (mainly children up to 12 years of age). A clear correlation between age and alpha-tocopherol concentration was also found, which the authors associated with higher cholesterol concentration in older age groups.

In the context of cardiovascular risk and safety of statins, it is important to relate the concentration of vitamin E to the current cholesterol concentration. The research by Spencer et al. [25] showed that in three different countries with different levels of cardiovascular disease risk (mortality rates of 212/100,000 in Finland, 145/100,000 in Northern Ireland, 43/100,000 in Italy), the concentration of vitamin E relative to cholesterol did not differ significantly. On the other hand, another study found that lipid-corrected vitamin E levels were more strongly correlated with the risk of death from ischemic heart disease than arterial blood pressure, cholesterol levels or the number of cigarettes smoked [42]. This variability may be due to the amount of vitamin E carriers in plasma, i.e. LDL.

A meta-analysis of six studies published in 2015 showed a decrease in plasma concentrations of CoQ10 during hypolipemic treatment with statins in adults [43]. In three of these studies, only men were evaluated, and only one study lasted longer than 12 weeks. The number of participants in placebo-controlled studies ranged from 9 to 32. The largest study involving 120 men with hypercholesterolemia did not include a control group, and none of the studies determined the molecular background of hypercholesterolemia. In addition, the results of studies concerning changes in the concentration of CoQ10 in muscle cells of patients treated with statins are contradictory [44, 45].

The inhibitory effect of statins on the synthesis of CoQ10 is particularly important due to the myotoxicity observed in some patients [46]. Muscle pain of varying severity affects up to 7% of patients treated [47]. Due to possible adverse metabolic consequences of this vitamin deficiency, supplementation attempts were made in adults. Early interventional studies in patients with skeletal muscle symptoms during statin therapy did not confirm the protective effect of dietary supplementation with this nutrient [21]. However, the conclusion of the last meta-analysis, published in 2018, suggests that supplementation with CoQ10 may reduce the severity of treatment-related adverse effects, although this requires further research [48].

There are only a few published studies on the concentration of CoQ10 in children with hypercholesterolemia. In 2002, Wittensetin et al. found no differences in plasma concentrations of this compound in 20 healthy children and 18 children with FH [49]. In an important study by Avis et al. from 2011, similar to adult studies, lower concentrations of CoQ10 were found in children with FH after treatment with statins versus before treatment [50]. This study assessed plasma concentrations of CoQ10 in peripheral blood mononuclear cells (PBMC) in children treated for at least 40 weeks with rosuvastatin doses increasing from 5 to 20 mg. Lower concentrations of CoQ10 were noted in plasma and PBMC, and no differences in mitochondrial ATP synthesis were found. Unfortunately, three patients with side effects were not included in the final analysis.

Our study presented no statistically significant differences in the concentration of CoQ10 between the groups of children with hypercholesterolemia who were and were not treated with statins. They also did not occur after the concentration of this compound was related to total cholesterol. There may be multiple reasons for the observed inconsistency in results. In adult studies, the presence of concomitant diseases compared to healthy (except for FH) children may be an important factor. It was shown that the concentration of CoQ10 decreased in the course of inflammatory diseases, hypertension, diabetes, periodontium diseases, depression and cancer, as well as in elderly people and athletes after great physical effort [14].

The lack of differences in CoQ10 concentration may also be influenced by individual sensitivity to changes in metabolic processes or the age-dependent efficiency of compensation mechanisms. In the analyzed subgroup of four girls treated with statins (Fig. 2), a clear difference is observed in the course of the curve of the first patient in comparison to the other three, in whom the CoQ10 concentrations, after a fairly significant increase in the first months of treatment, return to baseline or slightly below baseline values. The metabolic intensity of the developing organism and its compensatory abilities are higher than in adults, and thus it is easier to maintain the oxidoreductive balance despite the blockage of the enzyme enabling the synthesis of CoQ10.

The duration of intervention may also be important, as the meta-analysis showed a greater effect of statins on the concentration of CoQ10 in studies lasting less than 12 weeks than in studies conducted over longer periods [43]. In a long-term study by Avis et al., a decreased concentration of CoQ10 was observed in children, but different doses of rosuvastatin were used [50].

Possible reasons for the observed differences between the results of the described studies and the results of our study may be the size of groups, different inclusion criteria and differences in study design. A number of studies in adult subjects described in the meta-analysis reported group sizes comparable to that in our study, but the clinical criteria for inclusion are less precise, and the cause of hypercholesterolemia is not exclusively FH. Wittenstein’s study meets the diagnostic criterion, but no treatment was introduced. The interventional study by Avis et al. utilizes values before the introduction of treatment as control values. Differences may also be due to the severity of disease. New studies conducted among children with type 1 diabetes showed higher concentrations of vitamin E and CoQ10 in those with metabolically unbalanced diabetes compared to those with good glycemic control and healthy children [51].

The main limitation of our study is the small size of the groups, but this results from the fact that FH is less frequently diagnosed in children than among adults. Strict inclusion criteria for the study and precise selection of the control group in terms of sex and age are advantages. Collaboration between centers and increasing the size of the groups will allow future studies to take into account factors such as the time of treatment initiation, the type of preparation or the dose necessary to achieve the expected LDL concentration. Determination of the concentration of antioxidant vitamins in the plasma in future studies may be extended by the assessment of their concentrations in PBMC. The use of drugs other than statins in the lipid-lowering treatment of children with FH will enable the design of studies in which it will be possible to answer the question of whether the differences in the influence of statins on the concentration of antioxidant vitamins, revealed in various previous studies, result from their direct action or indirectly from the reduction in cholesterol and its carrier concentration. In order to assess the redox homeostasis of children with FH, it would be interesting to evaluate enzymatic and other non-enzymatic antioxidants as well as protein, lipid and DNA oxidation products.

Currently, the results of intervention studies among adults do not clearly confirm the need for supplementation. It is likely, however, that high doses of antioxidant vitamins used in adulthood do not compensate for faulty diet and lack of other healthy behaviors. It is possible that vitamins evaluated in the observational studies are only markers of consumption of products rich in other antioxidants and compounds influencing the course of cardiovascular diseases [52]. However, the possibility that patient age and severity of lesions are important factors for the intervention cannot be excluded. Taking into account the stages of atherosclerotic plaque formation and the intensity of oxidative processes in LDL, it is possible to conclude that intervention may have prophylactic significance only at an early stage of atherosclerosis development, because it activates compensatory mechanisms which, when appropriately programmed in the developing body, may constitute a protective mechanism for the rest of life according to the theory of “metabolic programming” [53, 54].

In the case of rare diseases, the greatest problem is the size of subject groups. Additional research is needed in groups of patients from different locations and taking into account different interfering factors. It is only the cumulative results of research evaluated, e.g. in meta-analyses, that make it possible to formulate recommendations. However, as there are no such summaries, we must use the data which are available as a basis for clinical management. Any information may be important in making decisions when, according to literature data, a large proportion of children with diagnosed FH are not treated due to concerns about the safety of the therapy. In our study, treatment with statins for 6 months did not significantly affect the concentration of antioxidant vitamins in the plasma of the examined children. We found no deficits of antioxidant vitamins in patients treated with statins, or any risk of adverse effects associated with an increase in their concentration. Patients with FH treated with statins are also managed with a low-fat and low-cholesterol diet that is very difficult to balance. To sum up, there is currently not sufficient evidence of the need for additional dietary modifications in terms of antioxidant vitamins.

## Electronic supplementary material

ESM 1 (XLSX 15 kb)

ESM 2 (DOCX 28 kb)

## Data Availability

Source data is available as supplemental files.

## Abbreviations

APCI: Atmospheric pressure chemical ionization
APO-B: Apolipoprotein B
CoQ10: Coenzyme Q10
DL: Desolvation line
FH: Familial hypercholesterolemia
HDL: High-density lipoprotein
HMG-CoA reductase: 3-Hydroxy-3-methylglutaryl-coenzyme A reductase
HPLC: High-performance liquid chromatography
LDL: Low-density lipoprotein
PBMC: Peripheral blood mononuclear cells
PXR: Pregnane X receptor
RBP4: Retinol-binding proteins
SD: Standard deviation
TRAP: Trapping antioxidant potential
TTR: Transthyretin
