# Supplementary material for: Effect of Statin Therapy on the Plasma Concentrations of Retinol, Alpha-Tocopherol and Coenzyme Q10 in Children with Familial Hypercholesterolemia
Source: Cardiovasc Drugs Ther. 2020 Oct 14;36(1):75–84. doi: 10.1007/s10557-020-07091-w (PMC8770382; doi:10.1007/s10557-020-07091-w)
Supplement: Supplementary file 2 — (DOCX 28 kb) [file 10557_2020_7091_MOESM2_ESM.docx]

**Supplementary data: Table 3**. Antioxidant vitamin levels in groups of examined children.

|  | **TREATED WITH STATINS** | | | | | **NOT TREATED WITH STATINS** | | | | | **P** |
| --- | --- | --- | --- | --- | --- | --- | --- | --- | --- | --- | --- |
|  | **mean** | **median** | **min** | **max** | **SD** | **mean** | **median** | **min** | **max** | **SD** |  |
| retinol  [μg/mL] | 0.02920 | 0.02540 | 0.01041 | 0.05596 | 0.01720 | 0.02603 | 0.03328 | 0.00465 | 0.04488 | 0.01489 | 0.613 |
| α-tocopherol  [μg/mL] | 0.93763 | 0.56521 | 0.17728 | 3.02874 | 0.85503 | 0.53398 | 0.53827 | 0.01257 | 1.32597 | 0.39546 | 0.362 |
| Q10 coenzyme  [μg/mL] | 0.18111 | 0.14766 | 0.08586 | 0.51040 | 0.11748 | 0.17411 | 0.16573 | 0.09665 | 0.26300 | 0.06365 | 0.605 |

**Supplementary data: Table 4**. Antioxidant vitamin levels in groups of examined children corrected by total cholesterol concentration.

|  | **TREATED WITH STATINS** | | | | | **NOT TREATED WITH STATINS** | | | | | P |
| --- | --- | --- | --- | --- | --- | --- | --- | --- | --- | --- | --- |
|  | **mean** | **median** | **min** | **max** | **SD** | **mean** | **median** | **min** | **max** | **SD** |  |
| retinol / total cholesterol | 0.00013 | 0.00011 | 0.00005 | 0.00026 | 0.00007 | 0.00010 | 0.00013 | 0.00002 | 0.00018 | 0.00006 | 0.389 |
| α-tocopherol / total cholesterol | 0.00425 | 0.00248 | 0.00087 | 0.01594 | 0.00418 | 0.00208 | 0.00240 | 0.00005 | 0.00460 | 0.00149 | 0.186 |
| Q10 coenzyme / total cholesterol | 0.00085 | 0.00062 | 0.00036 | 0.00269 | 0.00062 | 0.00068 | 0.00065 | 0.00035 | 0.00104 | 0.00023 | 0.832 |
